# Predicting cognition after subthalamic Deep Brain Stimulation in Parkinson’s Disease

**DOI:** 10.1038/s41531-025-01128-3

**Published:** 2025-08-28

**Authors:** Dorothee Kübler-Weller, Heiner Stuke, Melanie Astalosch, Luísa Martins Ribeiro, Elias Landfried, Gerd-Helge Schneider, Katharina Faust, Patricia Krause, Jan Roediger, Stefan Haufe, Mahta Mousavi, Bassam Al-Fatly, Claudia Spies, Friedrich Borchers, Andrea A. Kühn

**Affiliations:** 1 Movement Disorder and Neuromodulation Unit, Department of Neurology and Experimental Neurology, Charité - Universitätsmedizin Berlin, corporate member of Freie Universität Berlin and Humboldt-Universität zu, Berlin, Germany; 2 Department of Psychiatry and Psychotherapy, Charité - Universitätsmedizin, Berlin, Germany; 3 Robert Koch-Institute, Centre for Artificial Intelligence in Public Health Research, Berlin, Germany; 4 Department of Neurosurgery, Charité - Universitätsmedizin, Berlin, Germany; 5 Department of Neurosurgery, University Hospital Düsseldorf, Düsseldorf, Germany; 6 Berlin Institute of Health at Charité - Universitätsmedizin, Berlin, Germany; 7 Berlin Center for Advanced Neuroimaging (BCAN), Charité - Universitätsmedizin, Berlin, Germany; 8 Technische Universität, Berlin, Germany; 9 Physikalisch-Technische Bundesanstalt, Berlin, Germany; 10 Department of Anesthesiology and Intensive Care Medicine, Campus Charité Mitte and Campus Virchow-Klinikum, Charité - Universitätsmedizin, Berlin, Germany; 11 Berlin School of Mind and Brain, Humboldt-Universität zu, Berlin, Germany; 12 Deutsches Zentrum für Neurodegenerative Erkrankungen, Berlin, Germany

**Keywords:** Neuroscience, Psychology, Medical research, Neurology

## Abstract

Cognitive deficits have a high impact on quality of life in Parkinson’s disease (PD). This study takes into account the multifaceted etiology of cognition to estimate the cognitive outcome after deep brain stimulation (DBS) surgery in PD. Clinical, neuropsychological, perioperative, neuroimaging- and laboratory-based risk factors for cognitive dysfunction were prospectively assessed prior to surgery in 57 patients (21 female; age 60.2 ± 8.2; disease duration 10.5 ± 5.9 years, preregistered 9 June 2019 at clinicaltrials.gov, NCT03982953). Elastic net regularized regression and leave-one-out cross-validation were used to fit a multivariable model with the Montréal Cognitive Assessment (MoCA) change one year after surgery as primary outcome. The backward span had the most robust association with the cognitive outcome (rho = 0.499, *p* < 0.001**; c = 0.302). We propose a post-hoc prediction model for cognition based on the baseline MoCA and backward span (R² = 0.50). After clinical validation, our short and easily applicable prediction model could improve informed therapeutic decision making.

## Introduction

Cognitive deficits in Parkinson’s disease (PD) are among the non-motor symptoms with the greatest impact on quality of life^1^. Impairments range from no or minimal cognitive decline up to severe dementia (PD-D)^2^ and estimation of cognitive trajectories is challenging^3^. Nevertheless, early and reliable prediction is essential for therapeutic decision making, especially when deep brain stimulation (DBS) surgery is evaluated as an escalation of therapy. PD patients that underwent DBS surgery can suffer from deficits in long-term memory, verbal fluency and executive functions^4,5^. The long-term incidence of PD-D in patients with DBS, however, is comparable to medical treatment and considered a part of PD rather than an effect of DBS^6,7^. Nevertheless, considerable and sustained cognitive deterioration after DBS surgery can evolve rapidly in some patients^8^.

Preexisting cognitive deficits and higher age are the main risk factors for cognitive decline following DBS surgery in PD^9^. For an estimate of the cognitive trajectory of individual PD patients after DBS surgery, however, many aspects like the phenotype and course of PD, but also surgery- and stimulation-related aspects must be considered^10^.

General factors influencing cognition include education, nutritional status, comorbidities, inflammation, depression and apathy^11^. Decreased CSF Amyloid β and increased TAU levels have been shown to be predictive of more rapid and severe cognitive deterioration^12^. Serum neurofilament light chain (NfL) has good predictive value for cognitive decline in PD as well^13^.

PD-specific factors potentially influencing cognition are disease duration, symptom severity, dopaminergic dose and impulsivity. Other than that, PD patients with heterozygote gene mutations encoding glucocerebrosidase (GBA) display a more aggressive phenotype with a higher prevalence and a faster progression to PD-D^14^.

Amongst anatomical characteristics, the volume of the cholinergic Ncl. basalis of Meynert (NBM) has been shown to be predictive of cognition in PD: Patients with lower-than-expected NBM volumes at baseline have a 3.5-fold greater risk of becoming cognitively impaired after five years^15^. This finding was extended by a retrospective voxel-based morphometry (VBM) study, which demonstrated that preoperative NBM volume also predicted cognitive decline one year after DBS^16^.

Perioperative factors also increase the risk for complications such as postoperative delirium (POD), which in turn increases the risk of long-term cognitive decline^17^. Also, the length and depth of surgery and sedation have negative influences on postoperative cognitive outcomes^18^.

To account for the multifaceted etiology of cognitive change, this prospective study comprehensively assessed PD patients prior to DBS surgery by detailed phenotyping regarding non-motor symptoms, imaging, dementia markers and genetics. These markers were used to predict cognitive outcomes after DBS aiming to improve clinical decision making and perioperative management for optimal long-term benefit.

## Results

### Study cohort

**Fig. 1:**
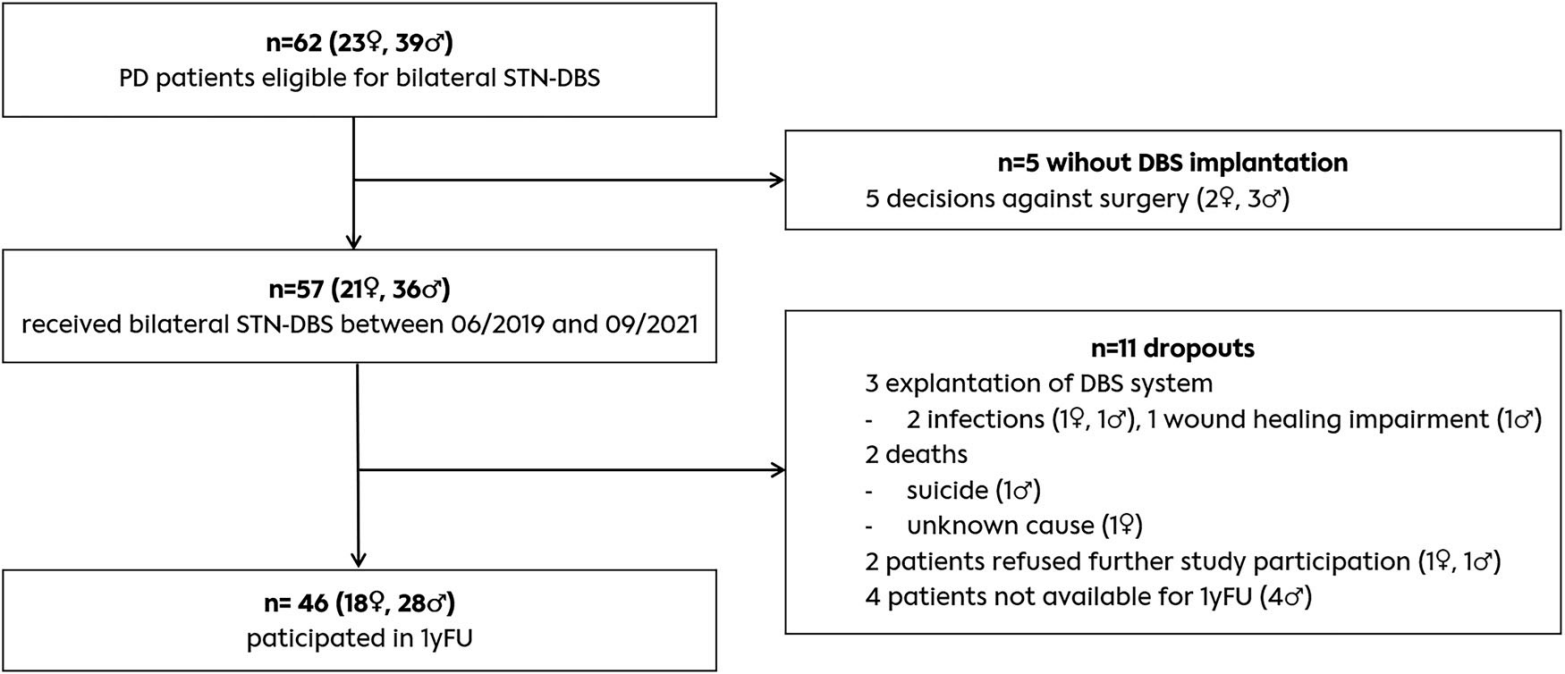
Study flow diagram. PD Parkinson’s disease, STN-DBS - deep brain stimulation in the subthalamic nucleus, 1yFU - Follow-up one year after DBS surgery.

62 PD patients evaluated as eligible for DBS surgery were included in the study. Five decided against invasive treatment.57 patients with PD received bilateral STN-DBS at our center between June 2019 and September 2021, 21 of them women (36.8%). As shown in the study flow diagram (Fig. 1), three patients had to be explanted during the study, two due to an infection of the device within the first 3 months after implantation and one due to prolonged wound healing impairment. Another two patients dropped-out due to suicide and death of unknown cause. Two patients withdraw study consent, and four patients were lost to FU. Hence, the data of 46 of the 57 patients during the study period was available for 1yFU.

Mean age of the cohort was 59.7 ± 8.2 and disease duration 10.6 ± 6.3 years. The results of all scales and scores assessed at preoperative baseline are shown in Table 1.

**Table 1:** Results of all assessed possible predictors of cognition one year after DBS-surgery (1yFU) reported as mean ± standard deviation (SD) with respective numbers (*n*) or percentages (%)

		*n* (%)	baseline mean ± SD	1yFU mean ± SD	Outcome global cognition at 1yFU		
					Multivariable coefficient c	Univariable coefficient rho	*p*
**General predictors**	**Sex**						
	Male	36 (63.2)			0	−0.259	0.082
	Female	21 (36.8)					
	**Age** (years)	57	60.2 ± 8.15		0	−0.246	0.099
	**Education level** (years)	57	15.4 ± 3.42		0	0.093	0.538
	**Burden of comorbidities** (CKI)	57	0.5 ± 0.73	0.5 ± 0.69	0	−0.095	0.532
	**Body Mass Index** (BMI)	57	27.1 ± 5.17		0	−0.018	0.907
	**Nutritional status** (MNA-SF)	57	12.2 ± 2.02	12.7 ± 1.7	0	−0.067	0.657
	**Depression screening** (BDI-II)	55	12.4 ± 8.01	13.1 ± 8.7	0	0.039	0.802
	**Apathy** (SAS)	54	14.9 ± 6.33	17.0 ± 6.5	0	−0.025	0.871
	**Need of support in activites of daily living** (ADL)	55	15.2 ± 12.42	12.5 ± 13.1	0	−0.059	0.699
	**Preoperative inflammation level** (CRP in μl)	57	2.1 ± 3.44		**−0.068**	−0.125	0.408
	**Blood serum neurofilament light chain** (NfL Z-Score)	21	0.9 ± 0.93		not included	−0.443	0.066
	**CSF dementia markers**						
	Phospho TAU (pg/ml)	23	36.4 ± 19.86		not included	−0.018	0.939
	Total TAU (pg/ml)	22	256.6 ± 138.89			−0.072	0.770
	Amyloid *β* 1-40 (pg/ml)	23	10755.09 ± 3519.97			0.158	0.506
	Amyloid *β* 1-42 (pg/ml)	22	958.1 ± 282.22			0.424	0.071
	Amyloid *β* 1-42 / 1-40 ratio	23	0.1 ± 0.02			**0.455**	0.044*
	Amyloid *β* 1-42 / total TAU ratio	19	4.3 ± 1.39			**0.501**	0.048*
**PD-specific predictors**	**Disease duration** (in years)	57	10.5 ± 5.87		0	−0.155	0.302
	**Motor predominance type**						
	Tremor-dominant	10 (17.5)			0	−0.183	0.224
	Akinetic-rigid	28 (49.1)			0	0.118	0.435
	Equivalent	19 (33.3)			0	0.029	0.848
	**Levodopa equivalent daily dose** (LEDD in mg)	57	1278.7 ± 341.03	514.3 ± 305.5	0	−0.047	0.757
	**Genetic background**						
	negative	43 (75.4)			0	0.077	0.618
	GBA	6 (10.5)			0	0.068	0.660
	LRRK2	4 (7.0)			0	−0.068	0.661
	PARK2	1 (1.8)			0	−0.260	0.088
	**Non-motor symptom severity** (MDS-UPDRS I)	55	10.0 ± 5.86	9.4 ± 5.8	0	0.091	0.553
	**Non-motor symptom severity** (MDS-UPDRS II)	55	11.9 ± 6.51	11.5 ± 7.7	0	−0.213	0.160
	**Motor symptom severity with medication** (MDS-UPDRS III MedON)	57	25.0 ± 12.29		0	−0.005	0.974
	**Motor symptom severity without medication** (MDS-UPDRS III MedOFF)	57	53.9 ± 15.32	StimON/MedOFF 27.1 ± 15.7 StimOFF/MedOFF 49.9 ± 16.7	0	0.028	0.853
	**Severity of fluctuations** (MDS-UPDRS IV)	52	7.6 ± 4.67	2.2 ± 2.5	**−0.110**	−0.148	0.344
	**Quality of life** (PDQ-39 PDSI in %)	56	32.6 ± 10.72		0	−0.041	0.789
	**Impulsivity** (QUIP-RS)	54	8.6 ± 10.37	6.6 ± 9.3	0	0.014	0.927
**Cognitive predictors**	**Impairment in classic neuropsychology test set** (number of deteriorated domains)	56	1.8 ± 1.34		**−0.196**	−0.163	0.286
	**CANTAB Connect**^**TM**^						
	**Attention and motor speed**						
	MOT: Reaction time (msec)	57	1019.0 ± 337.11	1064.6 ± 216.39	0.043	0.194	0.196
	RTI: Reaction time with 1 stimulus (msec)	57	381.4 ± 64.51	410.10 ± 88.48	0	0.003	0.985
	RTI: Reaction time with 5 different stimuli (msec)	57	436.1 ± 72.84	475.04 ± 101.43	0	−0.043	0.779
	**Visual learning**						
	PRM: Correct patterns immediate recall (%)	57	85.4 ± 11.59	82.3 ± 12.38	0	−0.083	0.583
	PRM: Correct patterns delayed recall (%)	54	73.7 ± 14.32	68.3 ± 16.32	0	−0.124	0.428
	PAL: Impairment associative learning (errors)	57	28.6 ± 18.48	26.6 ± 19.71	0	0.053	0.727
	PAL: Correct pairs (number)	57	8.2 ± 4.46	9.4 ± 5.06	0	0.015	0.923
	**Memory and working memory**						
	SSP: Items in correct sequence	56	5.2 ± 1.24	5.2 ± 0.93	**0.042**	0.283	0.059
	SSP: Items in correct backward sequence	56	5.1 ± 1.11	4.9 ± 0.80	**0.302**	**0.499**	<0.001**
	VRM: Words remembered in free recall	57	3.9 ± 2.93	4.1 ± 2.47	0	−0.154	0.306
	VRM: Words identified in immediate recognition	57	28.0 ± 4.15	27.7 ± 4.26	0	−0.271	0.068
	VRM: Words identified in delayed recognition	55	26.9 ± 4.30	27.2 3.99	0	−0.160	0.295
	**Executive functions**						
	MTT: Impairment in multitasking (errors)	57	16.8 ± 17.84	15.2 ± 17.53	0	−0.114	0.449
	MTT: Multitasking time (msec)	57	843.1 ± 170.59	900.3 ± 178.80	**−0.144**	−0.190	0.206
	MTT: Incongruency costs (msec)	57	89.1 ± 69.77	88.5 ± 76.79	**0.012**	0.102	0.499
	MTT: Multitasking costs (msec)	57	305.2 ± 169.73	285.9 ± 198.46	0	−0.007	0.965
	SWM: Repetitive errors	57	9.5 ± 1.83	18.9 ± 8.90	0	−0.254	0.089
	SWM: Strategy (score)	57	19.3 ± 7.36	6.13 ± 4.5	0	−0.074	0.625
	**Social cognition**						
	ERT: Emotion recognition ability	57	23.5 ± 5.81	22.4 ± 5.44	0	0.074	0.627
	**Theory of Mind (ToM) Yoni Task**						
	Cognitive ToM (% correct)	39	77 ± 19		0	0.155	0.390
	Affective ToM (% correct)	39	75 ± 22		0	0.012	0.946
	Control condition (% correct)	39	76 ± 21		0	0.081	0.655
**Imaging parameters**	**Volume of Ncl. basalis Meynert** (cm^3^)	52	0.777 ± 0.088		0	0.125	0.423
	**Volume of Ch1-3** (cm^3^)	52	0.879 ± 0.107		0	0.250	0.106
**Peri-operative predictors**	**EEG parameters**						
	Alpha Peak	40	9.1 ± 1.95		0	0.002	0.990
	Spatial edge frequency (SEF 95%)	40	25.2 ± 1.89		0	−0.195	0.292
	Burst suppression ratio	10	0.004 ± 0.01		not included	−0.302	0.098
	**Doses of sedatives**						
	**Surgery 1**						
	Propofol (mg/kg body weight)	43	31.9 ± 16.75		0	−0.064	0.722
	Remifentanil (µg/kg body weight)	51	77.1 ± 59.61		0	−0.071	0.665
	Fentanyl (µg/kg body weight)	3	37.9 ± 60.09		not included	−1.000	-
	**Surgery 2**						
	Propofol (mg/kg body weight)	56	13.2 ± 7.42		0	0.003	0.983
	Remifentanil (µg/kg body weight)	27	30.9 ± 17.41		not included	−0.209	0.337
	Fentanyl (µg/kg body weight)	31	5.6 ± 1.58		not included	−0.029	0.894
	**Dopaminergic medication pause** (hours)	57	15.8 ± 6.38		0	−0.002	0.991
	**Duration of surgery 1** (minutes)	57	360.6 ± 47.26		0	−0.076	0.618
	**Duration of surgery 2** (minutes)	56	115.7 ± 30.91		0	−0.130	0.396
	**Duration of postoperative delirium** (days)	57	7.5 ± 6.4		**−0.211**	−0.117	0.439
	**Severity of postoperative delirium** (Nu-DESC points)	57	2.8 ± 1.0		0	−0.064	0.672

Predictive values are reported as coefficients c from the multivariable regularized regression model and additionally from univariable Spearman correlations (rho) with their respective uncorrected *p*-values. *p < 0.05 (two-sided, uncorrected) **p < 0.005 (two-sided, uncorrected). c and/or rho are printed in bold if they contributed significantly to the respective model.

*CKI* charlson comorbidity index, *MNA-SF* mini nutritional index-short form, *BDI-II* beck depression inventory-version 2, *SAS* starkstein apathy scale, *CRP* C-reactive protein, *MDS-UPDRS* Unified Parkinson’s Disease Rating Scale by the Movement Disorder Society, *PDQ-39* Parkinson’s Disease Questionnaire, *PDSI* Parkinson’s Disease Summary Index, *QUIP-RS* Questionnaire for Impulsive-Compulsive Disorders in Parkinson’s Disease–Rating Scale, *MOT* Motor Screening Task, *RTI* Reaction Time test, *PRM* Pattern Recognition Memory test, *PAL* Paired Associates Learning task, *SSP* Spatial Span task, *VRM* Verbal Recognition Memory test, *MTT* Multitasking Test, *SWM* Spatial Working Memory test, *ERT* Emotion Recognition task, *Nu-DESC* Nursing Delirium Screening Scale.

### General outcome parameters

As expected, patients improved significantly with DBS concerning motor symptoms (MDS-UPDRS III *p* < 0.001), fluctuations (MDS-UPDRS IV *p* < 0.001), LEDD reduction (*p* < 0.001) and need of support in ADL (*p* = 0.020). UPDRS I (*p* = 0.694), UPDRS II (*p* = 0.285), nutritional status (MNA-SF *p* = 0.377), quality of life (PDQ-39 PDSI *p* = 0.081), symptoms of depression (BDI-II *p* = 0.938), apathy (SAS *p* = 0.409) and impulsivity (*p* = 0.378) did not show significant differences between preoperative and 1yFU assessment. Scores from baseline and 1yFU (where applicable) are displayed in Table 1.

There was no significant MoCA change postoperatively in comparison to the preoperative baseline (absolute MoCA: baseline 25.6 ± 3.0, 1yFU 25.1 ± 3.6; *p* = 0.285) but the cognitive outcome varied between patients (MoCA change: −0.5 ± 2.9). Within the CANTAB Connect^TM^ test set, the following parameters changed significantly: Reaction time with 1 (*p* = 0.011) and 5 stimuli (*p* = 0.001) from the RTI was longer, the backward span from the SSP was shorter (*p* = 0.019), multitasking time from the MTT was longer (*p* = 0.009), the strategy score from the MTT was smaller (*p* < 0.001) and emotion recognition was less (*p* = 0.002) at 1yFU in comparison to baseline.

### Results from multi- and univariable analysis

The multivariable analysis yielded a significant correlation between the true and predicted cognitive outcome (MoCA score difference at 1yFU in comparison to baseline, rho = 0.376, *p* = 0.010, mean absolute error = 2.200, root mean squared error = 2.747). All results from the multivariable (coefficients c) and univariable analyses (coefficients rho and respective *p*-values) can be found in Table 1.

**Fig. 2:**
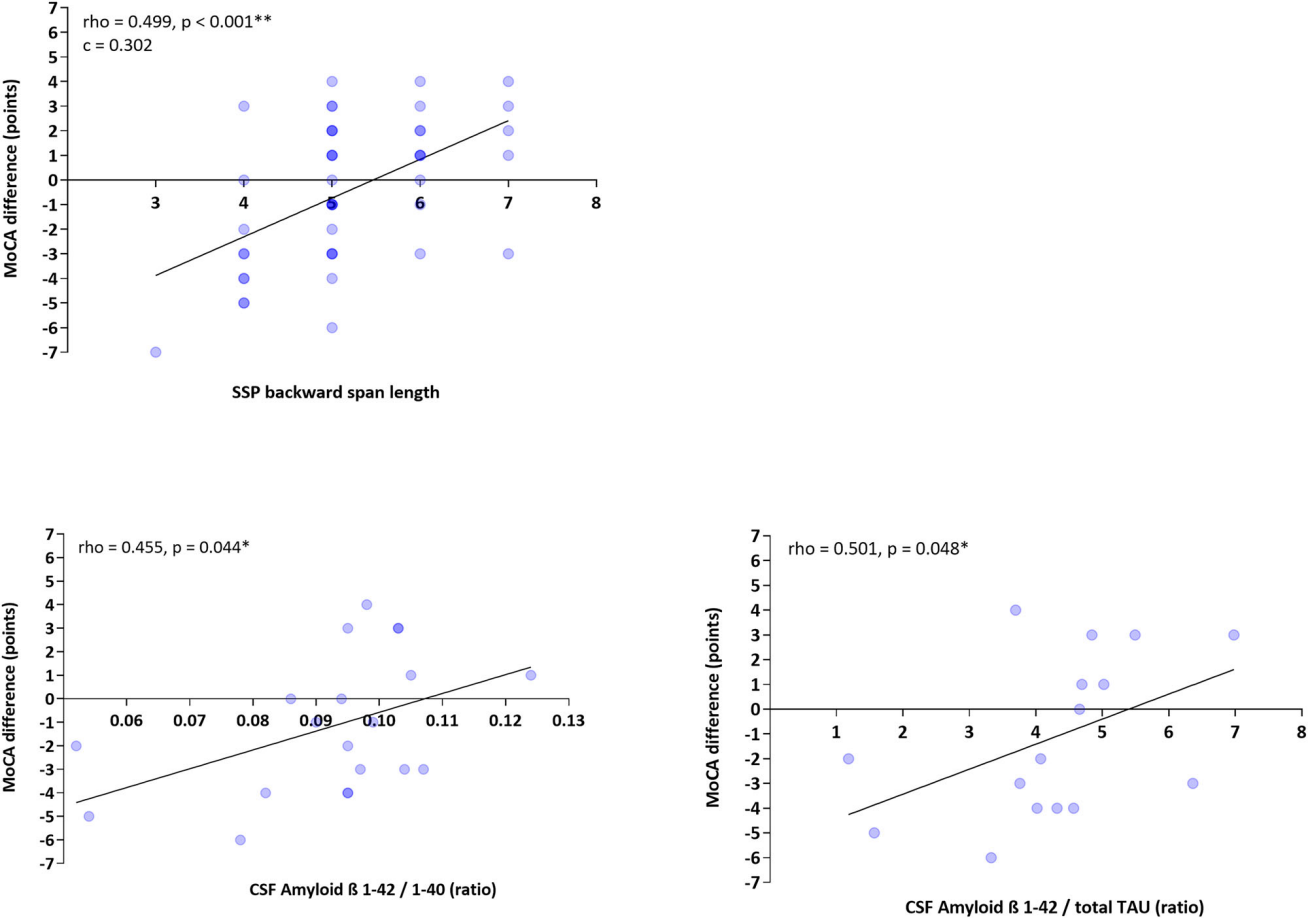
Scatterplots of statistically significant univariable correlations according to Spearman between predictors (x-axis) and the difference in MoCA points between the preoperative baseline and 1yFU. MoCA Montréal Cognitive Assessment, SSP Spatial Span task, rho correlation coefficient according to Spearman with respective *p*-value, c - correlation coefficient from multivariable model.

Visuospatial memory and working memory as measured by the backward span of the SSP, was a strong predictor of the cognitive outcome and was the only predictor that was associated with cognitive change one year after surgery in both the univariable and the multivariable analysis (rho = 0.499, *p* < 0.001**; c = 0.302). Scatterplots of these correlations can be found in Fig. 2.

Higher ß Amyloid β 1-42 / 1-40 and Amyloid β 1-42 / total TAU ratios were associated with better cognitive outcomes in the univariable model (rho = 0.455, *p* = 0.044* and rho = 0.501, *p* = 0.048*, respectively).

The inflammation level measured by means of the preoperative CRP from blood serum was negatively associated with the cognitive outcome one year after surgery in the multivariable analysis (rho = −0.125, *p* = 0.408, c = −0.068). In terms of PD-specific predictors, the severity of fluctuations assessed by the MDS-UPDS IV was related to worse cognitive outcomes in the multivariable analysis (rho = −0.148, *p* = 0.344; c = −0.110). In addition to the highly predictive backward span, the number of deteriorated cognitive domains in the classic neuropsychology test set (rho = −0.163, *p* = 0.286; c = −0.196), the length of the forward spatial span of the SSP testing visuospatial memory (rho = 0.283, *p* = 0.059; c = 0.042) and lower multitasking time (rho = −0.190, *p* = 0.206; c = −0.144) as well as higher incongruency costs (rho = 0.102, *p* = 0.499; c = 0.012) for conflicting stimuli in the MTT trials were associated with better cognitive outcomes in the multivariable analysis. Finally, of the perioperatively assessed data, the duration of POD was associated with a negative cognitive outcome in the multivariable analysis (rho = −0.117, *p* = 0.439; c = −0.211).

### Post-hoc model

The backward span of the SSP emerged as the strongest predictor of the cognitive outcome in the multivariable analysis and also showed a statistically significant univariable correlation with the cognitive outcome, suggesting that it is a strong predictor of cognitive outcome at 1yFU. To design a sparse and clinically applicable model for prospective evaluation, we performed a post-hoc analysis predicting the MoCA score at 1yFU using only the backward span and the preoperative MoCA score. The benchmark model including only the preoperative MoCA explained 27% of the variance in 1yFU-MoCA (R² = 0.27) in leave-one-out cross-validation. Adding the backward span of the SSP substantially improved model performance, increasing the explained variance to 50% (R² = 0.50) with a mean absolute error of 1.9 points. For clinical applicability, we rounded the multivariable coefficients to one decimal place yielding the following simplified linear model:

MoCA_₁yFU_ = 0.7 × MoCA_baseline_ + 1.7 × backward span_baseline_−1

**Fig. 3:**
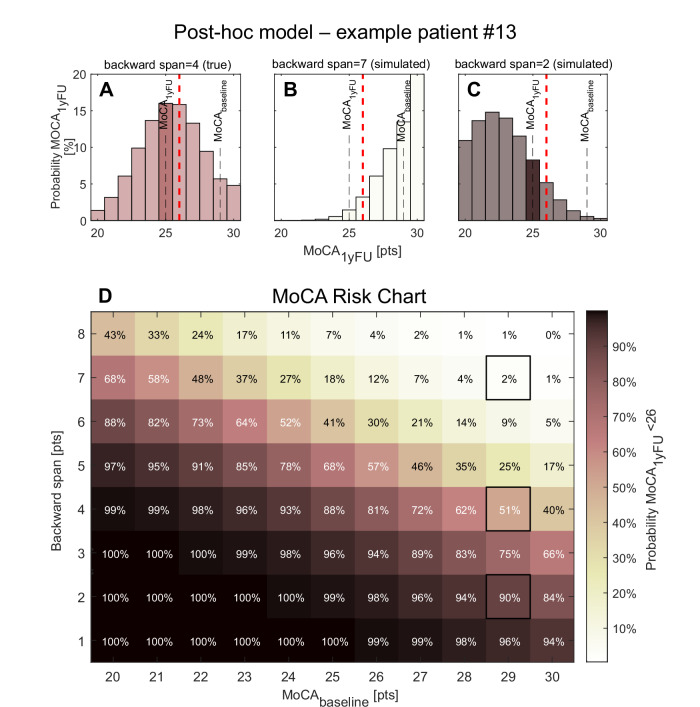
Post-hoc model for the prediction of cognition at 1yFU with MoCA +/− backward span from the SSP. **A** shows the predicted probability distribution of the MoCA score at 1yFU for a representative patient (#13) using the baseline MoCA score (29) together with the actual backward span (4), based on the sparse post-hoc model. **B**, **C** demonstrate how this distribution shifts toward better or worse cognitive outcomes when simulating higher (7) or lower (2) backward span performance, respectively. Predictions were obtained using the predict function in MATLAB R2024a, assuming Gaussian error and right-censoring at 30 points. Applying a MoCA cutoff of 26 points to differentiate between normal and impaired cognitive functioning, these simulations correspond to probabilities of 51% (backward span = 4), 2% (backward span = 7), and 90% (backward span = 2) that this patient would be classified as cognitively impaired at 1yFU, highlighting the strong influence of backward span performance on the predicted risk of postoperative impairment. **D** shows a discrete grid representation of the model predictions across the full range of baseline MoCA scores (20–30) and backward span values (1-8). Each cell represents the predicted probability that a patient with the corresponding combination will score below 26 on the MoCA at 1yFU. Darker shades indicate lower probabilities while lighter shades indicate higher probabilities. Percentage labels are shown within each cell. This visualization supports practical application of the model in clinical settings for individual risk estimation.

Figure 3 illustrates the clinical relevance of the model.

## Discussion

In this prospective cohort study, we found that in PD, the most robust predictor for the cognitive outcome one year after DBS surgery was the backward span. This test assesses visuospatial memory in combination with working memory, a set of skills mainly based on temporal and posterior cortical structures typically involved in Alzheimer’s disease with predominantly cholinergic underpinning^19^. Consistent with our results, a retrospective study in PD patients by Abboud and colleagues found a trend towards less favorable functional outcomes after 6 to 12 months in patients with visuospatial impairment before DBS surgery^20^. Preoperative deficits in visuospatial skills, verbal memory and language processing were also linked to cognitive complaints after DBS surgery reported by patients and/or their caregivers in a study by Mills and collegues^21^. However, we could not reproduce an influence of other classical cholinergic domains like learning and memory on postoperative cognition our study. In the case of the backward span, the memorized spatial sequence must additionally be manipulated in working memory recruiting fronto-striatal circuitry which suggests an additional involvement of dopaminergic structures. Also, in a recent review, both executive dysfunction and memory deficits, present prior to surgery were associated with worse cognitive outcomes after STN-DBS in PD patients^22^. Mana and colleagues, on the other hand, have shown executive dysfunctions to be most predictive of cognitive decline after DBS-surgery in PD in their recently published retrospective study^23^. Of note, the studies by Abboud^24^ and Mills^21^ did not test predictors equivalent to the backward span. Mana^23^ included a similar task, the Corsi Block task (SS-B), which did, however, not meet their criteria for a reliable predictor. In our opinion, the backward span should not only be part of further studies because of its predictive value but also because this test seems relatively sensitive in detecting change in working memory abilities^25^. This capacity might play a role in the design of future therapeutic studies on cognitive training in PD patients, especially with subtle cognitive deficits in early stages of the disease.

Higher Amyloid β 1-42/1-40 and Amyloid β 1-42/total TAU ratios from the CSF were associated with better cognitive outcomes. This constellation is known to be predictive for the development of Alzheimer’s disease and has lately also been studied in PD. This is the first study investigating the cognitive prognosis in PD patients after DBS taking into consideration biomarker profiles from CSF. In patients willing to undergo lumbar puncture, these findings could help refine estimation of the cognitive trajectory.

Other predictors also contributed significantly to the main multivariable model stressing the multifaceted influences on cognition in PD: The preoperative serum inflammation level, severity of motor fluctuations, deterioration in the classic neuropsychology test set and multitasking time were negatively associated whereas the forward spatial span and multitasking incongruency costs were positively associated with cognition one year after surgery. These findings stress the relevance of differential neuropsychological testing for the prediction of future cognitive decline. Apart from the SSP, the Stroop-like MTT assessing executive functions including set-shifting and suppression of premature responses seems to have a predictive potential of global cognition at 1yFU.

POD duration was also associated with a negative cognitive outcome one year after surgery in the multivariable analysis. Although in a strict sense, POD is not a true predictor as it occurs only postoperatively, this finding strengthens the importance of POD for the long-term cognitive outcome. As both cognitive deterioration and POD share most of their risk factors, there is a logic connection but we are the first to show that in a prospective study.

In contrast to a previous study from our group^16^, NBM volume did not contribute significantly to the model. Although data quality (retrospective study in 55 vs. prospective study in 57 PD patients undergoing DBS surgery) speaks in favor of the current project, the sample size of the current study is probably too small to assess NBM atrophy. As hypometabolism precedes atrophy, further studies could implement [^18^F]-FDG of the NBM to enhance prediction^26,27^.

Our cohort is representative for PD patients undergoing DBS surgery by age, disease duration, response to DBS etc. However, neither age nor the burden of comorbidities showed a significant impact on the cognitive outcome in our cohort. This finding may be due to a preselection of PD patients before they present for an evaluation of DBS surgery. The missing effect of the genetic background on the cognitive outcome after DBS, a current matter of debate^28–30^, could be due to the small sample size (6 patients with GBA-mutations) and should be interpreted with caution.

To facilitate clinical application, we derived a practical and sparse post-hoc model using only the baseline MoCA score and the backward span from the SSP as predictors of the cognitive outcome one year after DBS. These two tests are quick to administer (approximately 10 and 4 min, respectively) and do not require medical staff, making them highly feasible for routine use. The resulting linear model showed good predictive performance and can be applied as follows:

Predicted MoCA at 1yFU = 0.7 × MoCA + 1.7 × backward span - 1 at baseline.

While this model needs to be validated in a prospective multicenter setting, it represents a promising first step to improve individualized risk stratification of PD patients undergoing DBS.

This study must be discussed in the light of its limitations: Cognition is complex and multifaceted, more so in PD after STN-DBS. It has been stressed that only a prospective assessment of combined predictors can substantially add to the existing literature^31^. Therefore, we assessed many influencing factors as we are convinced that a reliable impression on the predictors of cognitive change can only be achieved if all the available evidence is considered. To counteract at the best possible rate the resulting shortcoming of the disproportion between patients and possible predictors, a sophisticated statistical design was applied to increase the reliability of the study results. Additionally, one must be aware of measurement error effects possibly inflating results that can play a substantial role in cognitive testing. Some variables could not be obtained from all patients and were subsequently excluded from the multivariable analysis and therefore have a low statistical validity. Another limitation is the relatively short follow-up period, so we are currently assessing this patient cohort five years after DBS surgery. A clear delineation of stimulation-related cognitive change is not possible with the current study.

Our results are expected to support evidence-based and personalized decision-making when advising PD patients considering STN-DBS. As a next step, our proposed slimmed down prediction model based on the MoCA and backward span of the SSP must be validated. Especially in combination with dementia markers from the CSF, the backward span has the potential as a fast and easily applicable predictor. In the future, we hope to contribute to the development of hypothesis-driven interventional trials, for example, on preventive strategies like cognitive training, with the goal to achieve optimal outcomes for every individual patient.

## Methods

**Fig. 4:**
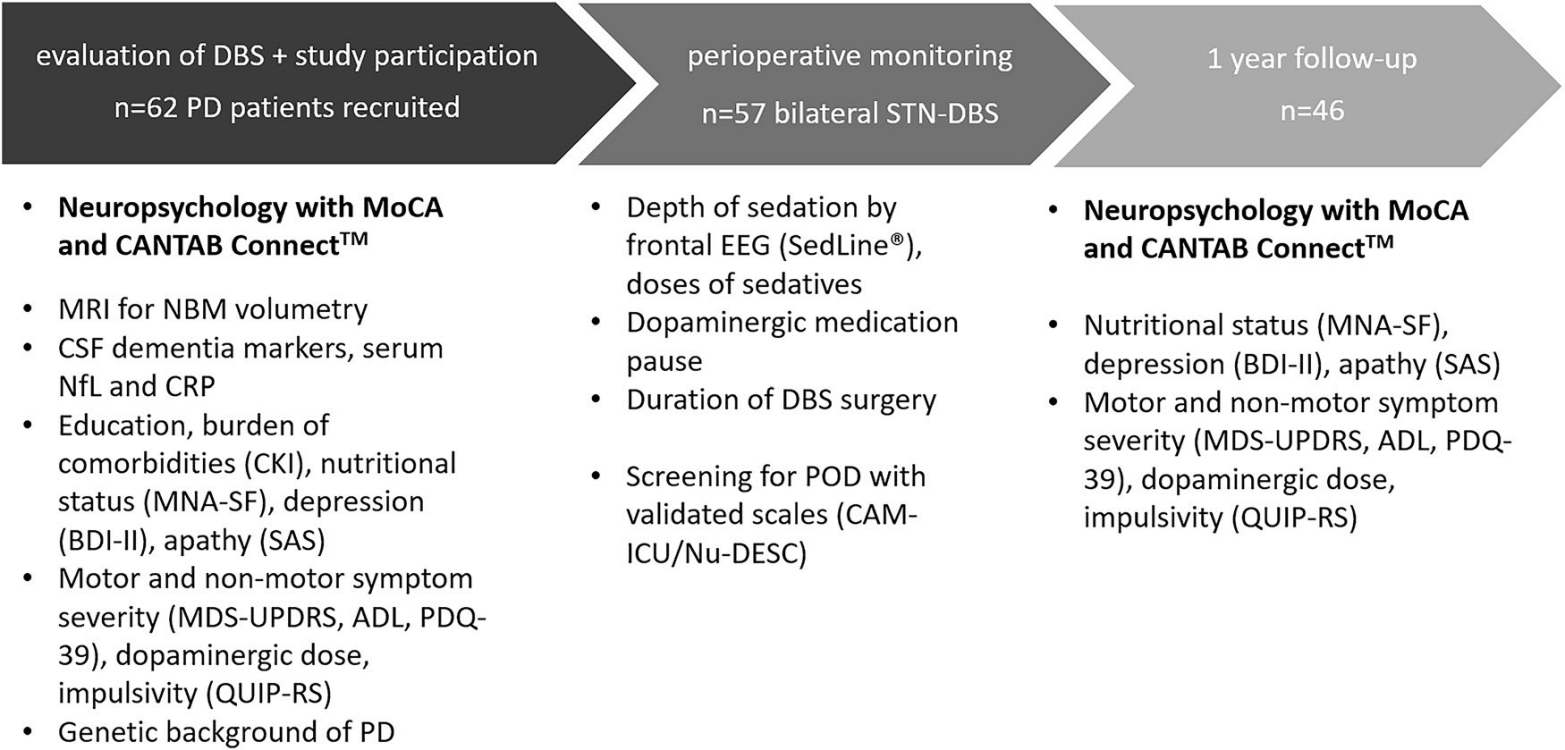
Schematic study overview. DBS deep brain stimulation, PD Parkinson’s disease, STN subthalamic nucleus, MoCA Montréal Cognitive Assessment, NBM Ncl. basalis of Meynert, NfL Neurofilament light chain, CRP C-reactive protein, CKI Charlson Comorbidity Index, MNA-SF Mini Nutritional Index - short form, BDI-II Beck Depression Inventory, SAS Starkstein Apathy Scale, MDS-UPDRS Unified Parkinson’s Disease Rating Scale by the Movement Disorder Society, ADL Need of support in activities of daily living, PDQ-39 Parkinson’s Disease Questionnaire, QUIP-RS Questionnaire for Impulsive-Compulsive Disorders in Parkinson’s Disease–Rating Scale, POD Postoperative delirium.

The study was registered at clinicaltrials.gov (NCT03982953, submitted 9 June 2019) and approved by the Charité ethics committee (EA2/040/19). TRIPOD guidelines were used for prediction model development and validation. Written informed consent was obtained from all patients. Study participation was proposed to all patients with the diagnosis of PD and the indication for bilateral DBS in the subthalamic nucleus (STN) after careful examination of eligibility for this treatment. Evaluation for the indication of DBS was conducted according to clinic-intern standard operation procedures that are in accordance with the guidelines for invasive therapies in PD^32^. The only exclusion criterium for study participation that did not apply with respect to a decision against DBS was a relevant language barrier. Figure 4 contains a study overview.

### Data from clinical routine

Within the clinical routine work-up during the evaluation process for DBS, the following clinical scales and questionnaires were applied and tested for their predictive value: Need of support in activities of daily living (ADL), symptom severity in PD motor and non-motor domains (MDS-UPDRS I-IV) including motor part MDS-UPDRS III with (MedON) and after wash-out of dopaminergic medication (MedOFF), quality of life (PDQ39), depression (BDI-II), apathy (SAS) and impulsivity (QUIP-RS). Motor predominance type, PD duration and levodopa equivalent daily doses (LEDD) were recorded. The genetic background of PD was assessed by testing for monogenetic causes of PD as well as susceptibility loci and genes^33^.

### General predictors of cognition

In addition to these clinical assessments, the following possible predictors of cognitive dysfunction after STN-DBS in PD were examined in the context of this prospective study: Years of education, nutritional status (BMI and MNA-SF). The burden of comorbidities (CKI) and the blood-based inflammation marker C-reactive protein (CRP) from the day before DBS surgery were assessed. In patients who agreed to undergo lumbar puncture (*n* = 23), total and phospho TAU as well as Amyloid β 1-40 and 1-42 was measured in the CSF. NfL, a specific marker for neuronal cell damage, was quantified from blood serum and Z-scores were calculated according to age and BMI^34^.

Besides the global cognitive screening Montréal Cognitive Assessment^35^ (MoCA), an extensive tablet-based cognitive test battery (CANTAB Connect^TM^) was applied prior to DBS surgery and at follow-up one year after surgery (1yFU). A description of the neuropsychological domains tested by different tests and their respective outcome measures can be found in Table 2 and in the supplementary material. Theory of Mind (ToM), the capacity to draw conclusions on other persons thoughts or emotions was investigated with the Yoni paradigm^36^ which includes an affective, cognitive and control condition. Outcomes measures are the percentages of correctly answered stimuli for each condition. Additionally, the number of domains rated as impaired in the standard neuropsychological test session during clinical evaluation for DBS surgery was assessed.

**Table 2:** Tabular overview of the neuropsychological domains assessed by the CANTAB Connect^TM^ test applied in the study with their respective content and outcome measures

Neuropsychological domain	Content of test	Measure (unit)
**Attention and motor speed**		
MOT: Motor Screening Task	Pretest, handling of the tablet	Mean reaction time (msec)
RTI: Reaction Time	Attention, movement and reaction time	Mean reaction time with 1 stimulus (msec)
		Mean reaction time with 5 different stimuli (msec)
**Visual learning**		
PRM: Pattern Recognition Memory	Visual Learning of abstract patterns	Correctly identified patterns in immediate recall (%)
		Correctly identified patterns in delayed recall (%)
PAL: Paired Associates Learning	Visual associative learning	Impairment in associative learning (errors)
		Correct pairs on first attempt (number)
**Memory and working memory**		
SSP: Spatial Span	Visuospatial memory	Visual short-time memory (items in correct sequence)
		Visual working memory (items in correct backward sequence)
VRM: Verbal Recognition Memory	Verbal learning and memory	Verbal learning (words remembered in free recall)
		Verbal learning (words identified in immediate recognition)
		Verbal learning (words identified in delayed recognition)
**Executive functions**		
MTT: Multitasking Test	Executive functions, set-shifting, suppression of premature responses	Impairment in multitasking (total errors)
		Multitasking time (msec)
		Incongruency costs for conflicting stimuli (msec)
		Multitasking costs for blocks with conflicting stimuli (msec)
SWM: Spatial Working Memory	Retainment of spatial information, strategy	Impairment in retaining spatial information (repetitive errors)
		Spatial working memory strategy (score)
**Social cognition**		
ERT: Emotion Recognition	Inference on basic emotional states	Emotion recognition ability (correct choices)

### Imaging

As imaging biomarkers, volumes of the cholinergic basal forebrain, i.e., its main nucleus, the NBM, and surrounding nuclei (Ch1-3) were assessed using VBM as previously described^37^. Basis were the preoperative structural MRIs with 3 Tesla acquired with Siemens Magnetom Vida or Skyra scanners. The T1-weighted gradient echo sequence was analyzed with MATLAB (version R2022a) by means of the CAT12 toolbox, an extension to the software SPM12. As partial brain volumes depend on total intracranial volume (TIV), the ratios NBM/TIV and Ch1-3/TIV were used for further calculations.

### Perioperative procedures

Perioperative procedures with a potential influence on cognitive outcomes were recorded. These include the duration of the perioperative pause of dopaminergic medication during electrode implantation (surgery 1) and doses of sedatives used during both surgeries (surgery 2 being the implantation of the impulse generator). Intraoperative sedation depth was measured with four EEG channels on the forehead (SedLine®). The power peak (averaged normalized power spectral density) and spectral edge frequency (SEF95) were calculated with EEGLAB^38^ running in MATLAB (version R2022a) as described in ref. ^37^. Burst suppression patterns were manually extracted and set in relation to the overall duration of surgery.

As postoperative delirium (POD) can lead to permanent cognitive deficits, screening for this complication was conducted with the CAM-ICU (Confusion Assessment Method for Intensive Care Unit)^39^ or Nu-DESC (Nursing Delirium Screening Scale)^40^ at non-ICU wards three times daily as previously described^37^. This subanalysis of the same cohort also contains a visualization of electrode localizations. Delirium severity (mean score of days with positive POD screening) and duration were calculated.

### Statistical analysis

Statistical analysis was carried out in IBM SPSS Statistics Version 28 and the sci-kit learn Version 1.3.0 in Python 3.9.1^41^. Descriptive statistics are reported as mean ± standard deviation (SD). Pre- and postoperative clinical scales and scores were compared by means of Wilcoxon signed-rank tests. The change in global cognition was calculated as the difference between MoCA points at 1yFU and baseline prior to surgery and will be referred to as “cognitive outcome” in the following.

The relationships between the predictor variables and the cognitive outcome were first analyzed with unadjusted Spearman correlation coefficients rho. Second, a multivariable prediction model was estimated to test the predictive performance of the combined predictor variables and to obtain adjusted multivariable coefficients (see below for details of the modeling approach). The resulting univariable and multivariable coefficients provide readers with complementary information. The univariable correlation coefficients indicate how strongly cognition changes after surgery depending on a single predictor variable (a negative correlation with age, for example, would indicate that older patients who have undergone surgery experience a stronger decline in cognition). The multivariable coefficient, on the other hand, indicates how strongly cognition changes in relation to a predictor variable when the influence of the other variables included in the model has already been taken into account. Thus, if a negative coefficient for a neuropsychological test is found in an age-adjusted model, this means that there is a greater deterioration in patients who score high on this neuropsychological test *for their age*, i.e., even when taking into account that older patients might generally score higher on this test. Both coefficients, therefore, provide complementary information and, as in previous studies^42^, we report both to provide as comprehensive a picture of the relationships as possible.

For the multivariable analysis, elastic net regularization^43^ was used due to the high number and multicollinearity of assessed predictors. This machine learning analysis mitigates overfitting and increases the stability of coefficient estimates and thus increases the statistical power and generalization ability of the model. It is recommended as it offers several advantages compared to traditional regression analyses^44^. Predictors with missing data in >50% of patients (CSF markers, NfL Z-scores, doses of Remifentanil and Fentanyl and burst suppression) were excluded from the multivariable analysis. To test the model’s predictive performance, leave-one-out cross-validation was conducted. In this approach, the model is repeatedly fitted to all but one patient (the training set) and is then applied to predict the cognitive outcome in the left out (test) patient. This procedure is repeated until all patients have been left out once. Hence, for each patient, there is a prediction of the cognitive outcome based on a model that was trained without using this patient’s data for training. The predictive performance of the model for test patients was assessed by calculating the mean absolute error and the root mean squared error as well as Spearman correlations between each patient’s predicted and true cognitive outcome at 1yFU. Each cross-validation fold comprised one-hot-encoding of categorical predictors, imputation of missing values using k-nearest-neighbors imputation as implemented in KNNImputer class of sklearn with default parameters^45^, z-standardization (subtraction of the sample mean and division by the standard deviation) of numerical variables, and training of the regression model in the training set. The hyperparameters of the elastic net regression model (regularization strength and ratio between L1 and L2 penalty) were optimized in a nested 20-fold cross-validation loop within each training set. A final multivariable model was fit with all patients and the hyperparameter set optimal in most cross-validation folds and its coefficients are reported as multivariable coefficients c.

Finally, based on these analyses, a concise post-hoc model to predict cognition one year after DBS surgery was developed and is proposed for clinical validation. To foster clinical applicability of this model, it only used the most predictive variable according to the multivariable analysis in addition to the preoperative MoCA baseline. While the main analysis focused on MoCA change to identify predictors of cognitive decline, the post-hoc model aims at clinical decision-making. Therefore, the absolute MoCA score at 1yFU was used as the outcome as it allows direct comparison to established thresholds for mild cognitive impairment in PD (MoCA <26) which is particularly relevant in the context of DBS eligibility evaluation. The model was implemented as a standard linear regression with Gaussian errors and the resulting prediction equation operates on the outcome scale (i.e., predicted MoCA score at 1yFU). The performance of this model was assessed using the coefficient of determination (R^2^) and compared to a benchmark model using only preoperative MoCA baseline as a predictor in a leave-one-out cross-validation fashion. Predicted probability distributions for Fig. 3 were generated using MATLAB R2024a with the fitlme function, using baseline MoCA and backward span as fixed effects. Predictions were obtained using the predict function with the name-value pair argument “Prediction”, “observation” to retrieve the expected value and associated 95% confidence intervals. The resulting normal distributions were right censored at the maximum MoCA value (30 points) to reflect scale boundaries. To visualize model predictions across the clinically relevant value ranges of baseline MoCA and backward span, we additionally created a discrete grid representation (Fig. 3D). For each combination of MoCA (20–30) and SSP backward span (1–8), the predicted probability of scoring below 26 at 1yFU was computed using the fitted linear model and displayed as a color-coded matrix with overlaid percentage labels. This representation could serve as a practical lookup tool to support individual risk stratification in clinical decision-making.

## Supplementary information

Supplementary_Methods

## Data Availability

Due to data privacy restrictions, patient datasets are not publicly available but can be provided by the corresponding author upon reasonable request.

## Code availability

The analysis code for the machine learning model, the post-hoc model and the generation of Fig. 3 can be found in the Supplementary Methods.
