# Supplementary material for: Predicting cognition after subthalamic Deep Brain Stimulation in Parkinson’s Disease
Source: NPJ Parkinsons Dis. 2025 Aug 28;11:265. doi: 10.1038/s41531-025-01128-3 (PMC12394401; doi:10.1038/s41531-025-01128-3)
Supplement: Supplementary file 1 — Supplementary_Methods [file 41531_2025_1128_MOESM1_ESM.pdf]

CANTAB Connect^{TM} cognitive test battery

The Motor Screening Task (MOT) serves as a pretest introducing and checking the handling of the tablet screen. In the Reaction Time test (RTI), patients must press buttons as fast as possible. Whereas in the simple trials, there is only one button that is always presented at the same location, five different buttons serve as cues in the advanced trials. The Pattern Recognition Memory test (PRM) assesses visual learning in an immediate and delayed recognition phase. Patients must identify a previously presented abstract pattern out of two choices. In the Paired Associates Learning task (PAL), visual learning is tested in combination with spatial memory. After different patterns have been displayed in different locations on the screen, patients must memorize the location of the pattern presented later during the trial. The Spatial Span task (SSP) is a digitalized version of the Corsi block span assessing visuospatial working memory. Patients are asked to remember and reproduce a spatial time sequence both in the same order as presented and backwards. The number of items in the longest correct sequence is called span. In the Verbal Recognition Memory test (VRM), verbal learning is assessed. There is a free recall condition without cues and an immediate and delayed recognition condition where every previously learned word must be discriminated from another word. In the Multitasking Test (MTT), conflicting stimuli must be integrated by shifting the focus of attention: Patients are asked point to either the side or the direction of an arrow presented at the screen while ignoring stimuli irrelevant to the task. In the Spatial Working Memory test (SWM), patients must find and memorize specific stimuli with an increasing number of possibilities. The Emotion Recognition task (ERT) measures the ability to infer emotions from facial expressions in a multiple-choice paradigm.

Code

Multivariable model (elastic net regression)

```
import numpy as np
import pandas as pd
import matplotlib.pyplot as plt
import seaborn as sns
import scipy.stats as stats
from sklearn.preprocessing import StandardScaler
from sklearn.linear_model import LogisticRegressionCV, LogisticRegression, ElasticNetCV,
ElasticNet, LinearRegression
from sklearn.model_selection import LeaveOneOut, GridSearchCV, KFold, StratifiedKFold
from sklearn.impute import KNNImputer
from sklearn.metrics import mean_absolute_error

rawtab=pd.read_excel('PD_DBS_Cognition.xlsx',header=1).set_index('subject')

y=rawtab['MOCA_diff']
y=y[~y.isnull()]
X=rawtab.loc[y.index,rawtab.columns.drop(['MOCA_diff','Burst_suppression'])]
binary=X.columns[X.nunique()==2]
X['sex']=X['sex']-1
categorical=['predominant_type','ROPAD']
for var in categorical:
    X=X.join(pd.get_dummies(X[var],prefix=var).astype(float)).drop(columns=var)
X.drop(columns=X.columns[X.columns.str.startswith('length_of_stay')],inplace=True)
binary=X.columns[X.nunique()==2]
numerical=list(set(set(X.columns.to_list())-set(binary)))

loo = LeaveOneOut()
regressors=[]
preds=np.empty(len(y))
for tri, tei in loo.split(X):
```

```
    print(tei)
    trX = X.iloc[tri,:]
    scaler1=StandardScaler().fit(trX[numerical])
    trX[numerical]=scaler1.transform(trX[numerical])
    imputer=KNNImputer().fit(trX)
    trX=pd.DataFrame(imputer.transform(trX))
    trX.columns=X.columns
    scaler2=StandardScaler().fit(trX[numerical])
    trX[numerical]=scaler2.transform(trX[numerical])
        reg=ElasticNetCV(cv=20,l1_ratio=[0.01,0.1,0.3,0.5,0.7,0.9],alphas=np.exp(np.arange(-
4,4,0.5)),random_state=0,max_iter=5000).fit(trX,stats.zscore(y.iloc[tri]))
    regressors.append(reg)
    teX=X.iloc[tei,:]
    teX[numerical]=scaler1.transform(teX[numerical])
    teX=pd.DataFrame(imputer.transform(teX))
    teX.columns=X.columns
    teX[numerical]=scaler2.transform(teX[numerical])
    preds[tei]=reg.predict(teX) * y.iloc[tri].std() + y.iloc[tri].mean()

hyperparams = pd.Series([(x.alpha_, x.l1_ratio_) for x in regressors])
hp_freq=hyperparams.value_counts()
stats.spearmanr(y,preds)
mean_absolute_error(y, preds)

def final_model(X,y,hyperparam,numerical):
    cols=X.columns
    X[numerical]=StandardScaler().fit_transform(X[numerical])
    X=pd.DataFrame(KNNImputer().fit_transform(X))
    X.columns=cols
    X[numerical]=StandardScaler().fit_transform(X[numerical])
     reg=ElasticNet(random_state=0,alpha=hyperparam[0],l1_ratio=hyperparam[1],max_iter=5
000).fit(X,stats.zscore(y))
    return reg
```

```
ax=sns.regplot(x=y,y=preds,ci=None)
ax.set_xlabel('True MOCA score change')
ax.set_ylabel('Predicted MOCA score change')
ax.set_box_aspect(1)

final_reg=final_model(X.copy(),y,hp_freq.index[0],numerical)
final_coef=pd.Series(final_reg.coef_.ravel())
final_coef.index=X.columns
```

Post-hoc model and generation of Figure 4

```
% This script performs linear modeling and prediction of cognitive outcomes
% (MoCA at 12 months) after STN-DBS using baseline MoCA and backward span (SSPRSL).
% It includes model fitting, LOOCV, probability distribution estimation,
% and visualization as in Figure 4 (A–D).

clear all; close all; clc;

MOCAthresh = 26;    % MOCA threshold for FigD

%% Load data from Excel
pth = "C:\\Users\\jan-r\\Downloads\\PD_DBS_Cognition_230627.xlsx";
Data1 = importfile1(pth, "PD_DBS_Cognition_230114 - final", [3, Inf]);
Data2 = importfile2(pth, "3 und 12MFU", [2, Inf]);

%% Combine and clean data
DataSparse = table(Data1.subject, Data2.MOCA_12, Data1.MOCA, Data1.SSPRSL);
DataSparse.Properties.VariableNames = {'subject','MOCA_12','MOCA','SSPRSL'};
DataSparse.SSPRSL([23,27]) = 5; % Mean imputation of missing values

%% Fit LOOCV model with predictors MoCA + SSP
fm = 'MOCA_12 ~ MOCA + SSPRSL';
fm_base = 'MOCA_12 ~ MOCA';
```

```
n = height(DataSparse);
yhat = nan(n,1);
yhat_base = nan(n,1);
CIy = nan(n,2);

for i = 1:n
    train = DataSparse;
    train(i,:) = [];
    lm = fitlme(train, fm);
    lm_base = fitlme(train, fm_base);
    [yhat(i), CItmp] = predict(lm, DataSparse(i,:), "Prediction", "observation");
    [yhat_base(i), ~] = predict(lm_base, DataSparse(i,:), "Prediction", "observation");
    CIy(i,:) = CItmp;
end

% Cap predictions at max MoCA score
yhat(yhat > 30) = 30;
yhat_base(yhat_base > 30) = 30;

%% Get posterior probability distributions (0–30 MoCA bins)
pred_pdf = predict_probabilities(yhat, CIy(:,1), CIy(:,2), 0:50);
pred_pdf(:,31) = sum(pred_pdf(:,31:end),2); % cap >30
pred_pdf(:,32:end) = [];

%% Report model performance
R2 = getCoD(yhat, DataSparse.MOCA_12);
disp(['Model R2: ', num2str(R2)]);

R2_base = getCoD(yhat_base, DataSparse.MOCA_12);
disp(['Model R2 benchmark: ', num2str(R2_base)]);

%% Compare full posthoc model to baseline model
lm_full = fitlme(DataSparse, fm);
lm_base = fitlme(DataSparse, fm_base);
```

```
compare(lm_full, lm_base);


%% Final combined panel figure (Figure 4A–D)
% Prepare data for panels A–C
SSPRSL_vals = [4, 7, 2];  % Panel A–C
titles = {'A', 'B', 'C'};
ptID = 13;
exampleData = DataSparse(ptID,:);
pdfs = zeros(31, 3);

for i = 1:length(SSPRSL_vals)
    exampleData.SSPRSL = SSPRSL_vals(i);
    [yhat_tmp, CI_tmp] = predict(lm_full, exampleData, "Prediction", "observation");
    pdf = predict_probabilities(yhat_tmp, CI_tmp(1), CI_tmp(2), 0:50);
    pdf(31) = sum(pdf(31:end)); pdf(32:end) = [];   % Cap at 30
    pdfs(:, i) = pdf;
end

% Prepare data for panel D
[X, Y] = meshgrid(20:30, 1:8);
tbl = table(X(:), Y(:), 'VariableNames', {'MOCA', 'SSPRSL'});
[yHat, yCI] = predict(lm_full, tbl, 'Prediction', 'observation');
matpdfs = nan(31,length(yHat));
for i = 1:length(yHat)
    pdf = predict_probabilities(yHat(i), yCI(i,1), yCI(i,2), 0:50);
    pdf(31) = sum(pdf(31:end)); pdf(32:end) = [];   % Cap at 30
    matpdfs(:, i) = pdf;
end
P = sum(matpdfs(1:MOCAthresh,:));
Z = reshape(P, size(X));

% Plot all panels
figure('Position', [100 100 1200 800]);
```

```
cmap = flipud(pink(256));
p_moca26 = sum(pdfs(1:MOCAthresh+1,:),1);
ylimhist = 0.2;
sgtitle(['Model prediction patient #',num2str(ptID)])
for i = 1:3
    subplot(3,3,i);

    % Identify color corresponding to Panel D
    color_idx = max(1, round(p_moca26(i) * 255) + 1);  % Ensure within 1–256
    this_color = cmap(color_idx, :);

    barh = bar(0:30, pdfs(:,i), 1, 'FaceColor', this_color, FaceAlpha=0.6);
    hold on;

    % Add cutoff at MOCAthresh (-0.5)
    line([MOCAthresh MOCAthresh], [0 ylimhist], 'Color', 'red', 'LineStyle', '--', 'LineWidth', 
1.5);

    % MOCA baseline (fixed across simulations)
    hx = xline(exampleData.MOCA,'--
','MOCA_{0}','Color',[0,0,0],'LabelHorizontalAlignment','center','LabelVerticalAlignment','to
p');
    hx.FontSize = 8;

    % Observed MOCA_12
    actual = DataSparse.MOCA_12(ptID);
    bar(actual, pdfs(actual+1,i), 1,'FaceColor', this_color, 'FaceAlpha', 1);
    hx = xline(actual,'--
','MOCA_{12}','Color',[0,0,0],'LabelHorizontalAlignment','center','LabelVerticalAlignment','t
op');
    hx.FontSize = 8;
    % Labels
    switch i
        case 1
```

```
            ylabel('Probability MOCA_{12} [%]');
            title(['SSPRSL=', num2str(SSPRSL_vals(i)),' (true)']);
        case 2
            xlabel('MoCA_{12} [pts]');
            title(['SSPRSL=', num2str(SSPRSL_vals(i)),' (simulated)']);
        case 3
            title(['SSPRSL=', num2str(SSPRSL_vals(i)),' (simulated)']);
    end
    ylim([0 ylimhist]); xlim([19.5 30.5]);
end

subplot(3,3,4:9);
imagesc(20:30, 1:8, Z);
colormap(cmap);
cb = colorbar;
cb.Ticks = 0:0.1:1;
cb.TickLabels = compose('%d%%', 0:10:100);
ylabel(cb, ['Probability MoCA_{12} <',num2str(MOCAthresh)],'Rotation', 90,'FontSize', 11,
'FontWeight', 'normal');
xlabel('MOCA_{0} [pts]'); ylabel('Backward span (SSPRSL) [pts]');
set(gca, 'YDir', 'normal'); xticks(20:30); yticks(1:8);

% Annotate grid values
for i = 1:8
    for j = 1:11
        val = Z(i,j);
        col = [1 1 1]*(val > 0.5);
        text(19+j, i, sprintf('%.0f%%', 100*val),...
            'HorizontalAlignment','center', 'Color', col, 'FontSize', 8);
    end
end
%% --- Helper Functions ---
```

```
function PDDBSCognition230627 = importfile1(workbookFile, sheetName, dataLines)
%IMPORTFILE Import data from a spreadsheet
%  PDDBSCOGNITION230627 = IMPORTFILE(FILE) reads data from the first
%  worksheet in the Microsoft Excel spreadsheet file named FILE.
%  Returns the data as a table.
%
%  PDDBSCOGNITION230627 = IMPORTFILE(FILE, SHEET) reads from the
%  specified worksheet.
%
%  PDDBSCOGNITION230627 = IMPORTFILE(FILE, SHEET, DATALINES) reads from
%  the specified worksheet for the specified row interval(s). Specify
%  DATALINES as a positive scalar integer or a N-by-2 array of positive
%  scalar integers for dis-contiguous row intervals.
%
%  Example:
%  PDDBSCognition230627 = importfile("C:\Users\jan-r\Downloads\PD_DBS_Cognition_230627.xlsx", "PD_DBS_Cognition_230114 - final", [3, Inf]);
%
%  See also READTABLE.
%
% Auto-generated by MATLAB on 24-Mar-2025 13:16:14

%% Input handling

% If no sheet is specified, read from PD_DBS_Cognition_230114 - final
if nargin == 1 || isempty(sheetName)
    sheetName = "PD_DBS_Cognition_230114 - final";
end

% If row start and end points are not specified, define defaults
if nargin <= 2
    dataLines = [3, Inf];
end
```

```
%% Set up the Import Options and import the data
opts = spreadsheetImportOptions("NumVariables", 65);

% Specify sheet and range
opts.Sheet = sheetName;
opts.DataRange = dataLines(1, :);

% Specify column names and types
opts.VariableNames = ["subject", "sex", "age", "Illness_duration", "predominant_type",
"level_of_Education", "LEDD", "CKI", "BMI", "MNA_SF", "ROPAD", "MOCA",
"neuropsychological_testing_domains_affected", "ADL", "MDS_UPDRS_I",
"MDS_UPDRS_II", "MDS_UPDRS_III_ON", "MDS_UPDRS_III_OFF",
"MDS_UPDRS_IV", "BDI_II", "Starkstein_Apathy_Scale", "QUIP_RS", "PDSI",
"CRP_pre_OP", "OP1_duration", "OP2_duration", "dopaminergic_medication_break",
"MOTML", "RTISMDRT", "RTIFMDRT", "PRMPCI", "SSPFSL", "SSPRSL", "ERTTH",
"PRMPCD", "VRMFRDS", "VRMIRTC", "MTTTIC", "MTTLMD", "MTTICMD",
"MTTMTCMD", "SWMBE468", "SWMS", "PALTEA", "PALFAMS", "VRMDRTC",
"cog_accuracy", "aff_accuracy", "phy_accuracy", "alpha_peak", "SEF95",
"Burst_suppression", "Propofol_dose1", "Remifentanyl_dose1", "Propofol_dose2",
"Ch4_TIV", "Ch1_3_TIV", "NFL_Serum", "PhospoTAU_pgmlab141019565Davor66",
"GesamtTAU_pgmlab141019404Davor370", "Amyloid140_pgml",
"Amyloid142_pgmlab141019599davor562", "AmyloidRatio4240",
"Amyloid142gesamtTAURatio", "NeurofilamentLightProt_pgml829"];
opts.VariableTypes = ["double", "double", "double", "double", "double", "double", "double",
"double", "double", "double", "double", "double", "double", "double", "double", "double",
"double", "double", "double", "double", "double", "double", "double", "double", "double",
"double", "double", "double", "double", "double", "double", "double", "double", "double",
"double", "double", "double", "double", "double", "double", "double", "double", "double",
"double", "double", "double", "double", "double", "double", "double", "double", "double",
"double", "double", "double", "double", "double", "double", "double", "double", "double",
"double", "double", "double", "double"];

% Import the data
```

```
PDDBSCognition230627 = readtable(workbookFile, opts, "UseExcel", false);

for idx = 2:size(dataLines, 1)
    opts.DataRange = dataLines(idx, :);
    tb = readtable(workbookFile, opts, "UseExcel", false);
    PDDBSCognition230627 = [PDDBSCognition230627; tb]; %#ok<AGROW>
end
end

function PDDBSCognition230627S1 = importfile2(workbookFile, sheetName, dataLines)
%IMPORTFILE Import data from a spreadsheet
%  PDDBSCOGNITION230627S1 = IMPORTFILE(FILE) reads data from the first
%  worksheet in the Microsoft Excel spreadsheet file named FILE.
%  Returns the data as a table.
%
%  PDDBSCOGNITION230627S1 = IMPORTFILE(FILE, SHEET) reads from the
%  specified worksheet.
%
%  PDDBSCOGNITION230627S1 = IMPORTFILE(FILE, SHEET, DATALINES) reads
%  from the specified worksheet for the specified row interval(s).
%  Specify DATALINES as a positive scalar integer or a N-by-2 array of
%  positive scalar integers for dis-contiguous row intervals.
%
%  Example:
%  PDDBSCognition230627S1 = importfile("C:\Users\jan-
r\Downloads\PD_DBS_Cognition_230627.xlsx", "3 und 12MFU", [2, Inf]);
%
%  See also READTABLE.
%
% Auto-generated by MATLAB on 24-Mar-2025 08:09:15

%% Input handling

% If no sheet is specified, read from 3 und 12MFU
```

```
if nargin == 1 || isempty(sheetName)
    sheetName = "3 und 12MFU";
end

% If row start and end points are not specified, define defaults
if nargin <= 2
    dataLines = [2, Inf];
end

%% Set up the Import Options and import the data
opts = spreadsheetImportOptions("NumVariables", 82);

% Specify sheet and range
opts.Sheet = sheetName;
opts.DataRange = dataLines(1, :);

% Specify column names and types
opts.VariableNames = ["subject", "Datum_3", "MOTML_3", "RTISMDRT_3",
"RTIFMDRT_3", "PRMPCI_3", "SSPFSL_3", "SSPRSL_3", "ERTTH_3", "PRMPCD_3",
"VRMFRDS_3", "VRMIRTC_3", "MTTTIC_3", "MTTLMD_3", "MTTICMD_3",
"MTTMTCMD_3", "SWMBE468_3", "SWMS_3", "PALTEA_3", "PALFAMS_3",
"VRMDRTC_3", "MOCA_3", "Datum_12ON", "MOTML_12ON", "RTISMDRT_12ON",
"RTIFMDRT_12ON", "PRMPCI_12ON", "SSPFSL_12ON", "SSPRSL_12ON",
"ERTTH_12ON", "PRMPCD_12ON", "VRMFRDS_12ON", "VRMIRTC_12ON",
"MTTTIC_12ON", "MTTLMD_12ON", "MTTICMD_12ON", "MTTMTCMD_12ON",
"SWMBE468_12ON", "SWMS_12ON", "PALTEA_12ON", "PALFAMS_12ON",
"VRMDRTC_12ONMFU", "LEDD_12", "ClavienDindo_12", "BDI_II_12",
"Starkstein_Apathy_Scale_12", "QUIP_RS_12", "ADL_12", "MDS_UPDRS_I_12",
"MDS_UPDRS_II_12", "MDS_UPDRS_III_STIM_ON_MED_OFF_12",
"MDS_UPDRS_III_STIM_ON_MED_ON_12",
"MDS_UPDRS_STIM_OFF_MED_OFF_12", "MDS_UPDRS_STIM_OFF_MED_ON_12",
"MDS_UPDRS_IV_12", "MOCA_12", "MOCA_Diff", "MOCA_rel_Diff", "MNASF_12",
"cog_accuracy_12", "aff_accuracy_12", "phy_accuracy_12", "Datum_12OFF",
"MOTML_12OFF", "RTISMDRT_12OFF", "RTIFMDRT_12OFF", "PRMPCI_12OFF",
```

```
"SSPFSL_12OFF", "SSPRSL_12OFF", "ERTTH_12OFF", "PRMPCD_12OFF",
"VRMFRDS_12OFF", "VRMIRTC_12OFF", "MTTTIC_12OFF", "MTTLMD_12OFF",
"MTTICMD_12OFF", "MTTMTCMD_12OFF", "SWMBE468_12OFF", "SWMS_12OFF",
"PALTEA_12OFF", "PALFAMS_12OFF", "VRMDRTC_12OFFMFU"];
opts.VariableTypes = ["double", "datetime", "double", "double", "double", "double", "double",
"double", "double", "double", "double", "double", "double", "double", "double", "double",
"double", "double", "double", "double", "double", "double", "datetime", "double", "double",
"double", "double", "double", "double", "double", "double", "double", "double", "double",
"double", "double", "double", "double", "double", "double", "double", "double", "double",
"double", "double", "double", "double", "double", "double", "double", "double", "double",
"double", "double", "double", "double", "double", "double", "double", "double", "double",
"double", "datetime", "double", "double", "double", "double", "double", "double", "double",
"double", "double", "double", "double", "double", "double", "double", "double", "double",
"double", "double", "double"];

% Import the data
PDDBSCognition230627S1 = readtable(workbookFile, opts, "UseExcel", false);

for idx = 2:size(dataLines, 1)
    opts.DataRange = dataLines(idx, :);
    tb = readtable(workbookFile, opts, "UseExcel", false);
    PDDBSCognition230627S1 = [PDDBSCognition230627S1; tb]; %#ok<AGROW>
end

end

function pdf = predict_probabilities(yHat, lCI, uCI, x)
    sigma = diff([lCI,uCI],1,2)/(1.96*2);
    pdf = normpdf(x, yHat, sigma);
end


function R2 = getCoD(yHat, y)
    nanind = isnan(y)|isnan(yHat);
```

```
    y(nanind) = []; yHat(nanind) = [];
    SSR = sum((yHat - y).^2);
    TSS = sum((y - mean(y)).^2);
    R2 = 1 - SSR/TSS;
end
```
